# 3D isotropic FastView MRI localizer allows reliable torsion measurements of the lower limb

**DOI:** 10.1186/s41747-025-00631-9

**Published:** 2025-09-09

**Authors:** Felix L. Herr, Natascha Hohmann, Christian Dascalescu, Boj Hoppe, Hannah Gildein, Verena Schäfer, Jens Ricke, Boris M. Holzapfel, Lennart Schröder, Nina Hesse, Jörg Arnholdt, Paul Reidler

**Affiliations:** 1 Department of Radiology, University Hospital, LMU Munich, Munich, Germany; 2 Department of Orthopaedics and Trauma Surgery, Orthopaedic Oncology, Musculoskeletal University Center Munich (MUM), University Hospital, LMU Munich, Munich, Germany

**Keywords:** Extremities (lower), Femur, Magnetic resonance imaging, Reproducibility of results, Tibia

## Abstract

**Abstract:**

Computed tomography (CT) and magnetic resonance imaging (MRI) are commonly used to assess femoral and tibial torsion. While CT offers high spatial resolution, it involves ionizing radiation. MRI avoids radiation but requires multiple sequences and extended acquisition time. We retrospectively evaluated whether a three-dimensional isotropic MRI localizer (FastView) could serve as a reliable and faster alternative. In this retrospective single-center study, 60 lower limbs from 30 patients, aged 27.1 ± 11.5 years (mean ± standard deviation), 19 females and 11 males, were assessed using both FastView and a dedicated MRI protocol. FastView (5 × 5 × 5 mm^3^ voxels) imaged the entire lower limb in 17.4 s compared to nearly 7 min for the dedicated protocol. Torsion angles were measured independently by two readers. Agreement between methods was evaluated using intraclass correlation coefficients (ICCs), Bland–Altman plots, and Pearson *R*². No significant differences in torsion values were found (all *p* > 0.305). Femoral (ICC: 0.91–0.96) and tibial (ICC: 0.91–0.94) torsion showed excellent inter-modality agreement. Inter-reader reliability was also high (ICC: 0.95–0.99). Correlation values confirmed strong agreement (*R*²: 0.891–0.963). FastView demonstrated accuracy comparable to the dedicated protocol, offering a fast, efficient, and radiation-free option for routine torsion assessment.

**Relevance statement:**

FastView MRI localizer offers a fast and resource-efficient method for assessing lower limb torsion, potentially replacing standard multisequence protocols in routine clinical practice.

**Key Points:**

- FastView MRI enables lower limb torsion measurements with full-limb coverage in under 20 s.
- Torsion angles from FastView and dedicated MRI showed no significant differences.
- Femoral and tibial ICCs between 0.91 and 0.96 confirm excellent inter-protocol agreement.
- Inter-reader agreement was consistently high across both protocols.
- FastView may replace multisequence MRI protocols in routine clinical torsion assessment.

**Graphical Abstract:**

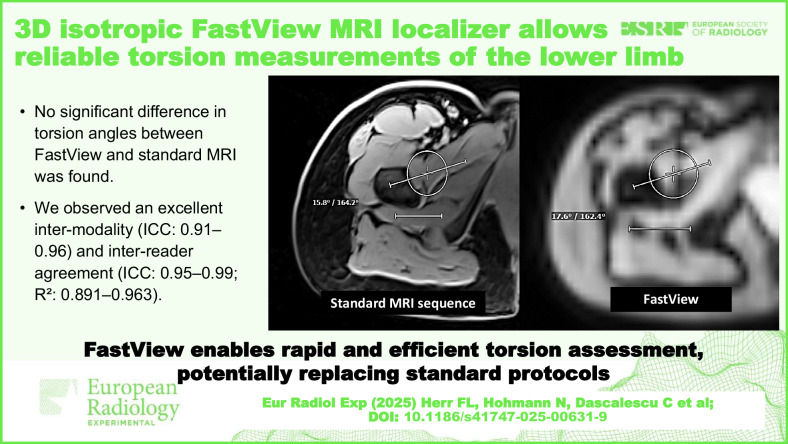

## Background

Accurate evaluation of femoral and tibial torsion plays a critical role in the diagnosis and treatment planning of various lower extremity disorders, such as patellofemoral instability, femoroacetabular impingement, and gait abnormalities [1, 2]. Historically, CT has been regarded as the imaging standard for these measurements, owing to its superior spatial resolution and high reproducibility [3, 4]. Nevertheless, the use of CT involves exposure to ionizing radiation, which is particularly concerning for younger patients and in situations that necessitate repeated imaging.

MRI offers a radiation-free alternative that also offers excellent soft-tissue contrast [5]. Several recent studies have demonstrated that MRI-based torsional assessments show good agreement with CT, supported by high interobserver consistency [6, 7]. However, traditional MRI-based torsion protocols are often lengthy and require multiple sequences to separately cover the hip, knee, and ankle joints. With the introduction of three-dimensional (3D) isotropic MRI techniques, there is growing potential to simplify and accelerate torsional imaging. The FastView localizer allows for rapid volumetric acquisition across the entire lower limb in a single scan, significantly reducing scan times and potentially enhancing clinical workflow efficiency [8]. However, the accuracy and reliability of torsion measurements derived from FastView images compared to dedicated MRI protocols remain to be fully elucidated.

The aim of this study is to determine whether torsion angles measured using the low-resolution 3D isotropic FastView MRI localizer are equivalent in accuracy and reproducibility to those obtained using a conventional dedicated MRI protocol. We hypothesize that FastView may serve as a time-saving and clinically viable alternative for lower limb torsion assessment.

## Materials and methods

This single-center retrospective study included 60 lower limbs from 30 patients, aged 27.1 ± 11.5 years (mean ± standard deviation), who underwent both a dedicated MRI torsion protocol and a 3D isotropic MRI localizer—FastView—acquisition from June 2022 to February 2025. The FastView localizer was acquired during continuous table movement and covered an 80-cm limb length with a spatial resolution of 5 × 5 × 5 mm^3^ voxels. The scan duration was 17.4 s. In contrast, the dedicated MRI torsion protocol consisted of three T1-weighted volumetric interpolated breath-hold examination (VIBE) sequences targeting the hips, knees, and ankles, with a total acquisition time of approximately 7 min, not including additional localizers and planning sequences. Technical details of the FastView and the dedicated protocols are shown in Table 1.

**Table 1:** Technical details of the FastView localizer and dedicated MRI sequences

	Voxel size (mm)	FOV (mm)	TE/TR (ms)	Flip angle (°)	Bandwidth (Hz/Pixel)	Table speed
FastView	5 × 5 × 5	420 × 480	1.44/2.56	0	801	4.6 cm/s
T1 VIBE pelvis/knee/ankle	1 × 1 × 1	549 × 230	2.46/6.3	10	302	NA

Device: MAGNETOM Vida, field strength: 3 T, software version: syngo MR XA60, Siemens Healthineers, Forchheim, Germany

Two independent radiologists measured femoral and tibial torsion angles on both protocols in separate reading sessions. Femoral torsion was evaluated using four established methods according to Lee et al [9], Reikeras et al [10], Tomczak et al [11], and Murphy et al [3]. Tibial torsion was assessed by measuring the angle between the posterior edge of the tibial plateau and the horizontal axis, as well as the angle between the centerline of the pilon tibiale-fibula and the horizontal axis. The sum of these two angles was defined as the tibial torsion. For comparison between protocols, the mean values of the two readers’ measurements were used. Inter-protocol and inter-reader reliability were assessed using intraclass correlation coefficient (ICC). In addition, Bland–Altman analysis and Pearson correlation coefficients (*R*²) were applied to evaluate the agreement between the dedicated protocol and the FastView localizer measurements. Normality of the data distributions was assessed using the Kolmogorov–Smirnov test. If both compared variables were normally distributed *(p* > 0.05), a two-sided paired *t*-test was applied. If normality was not met in either group, a Wilcoxon signed-rank test was used instead. A *p*-value < 0.05 was considered statistically significant.

## Results

**Fig. 1:**
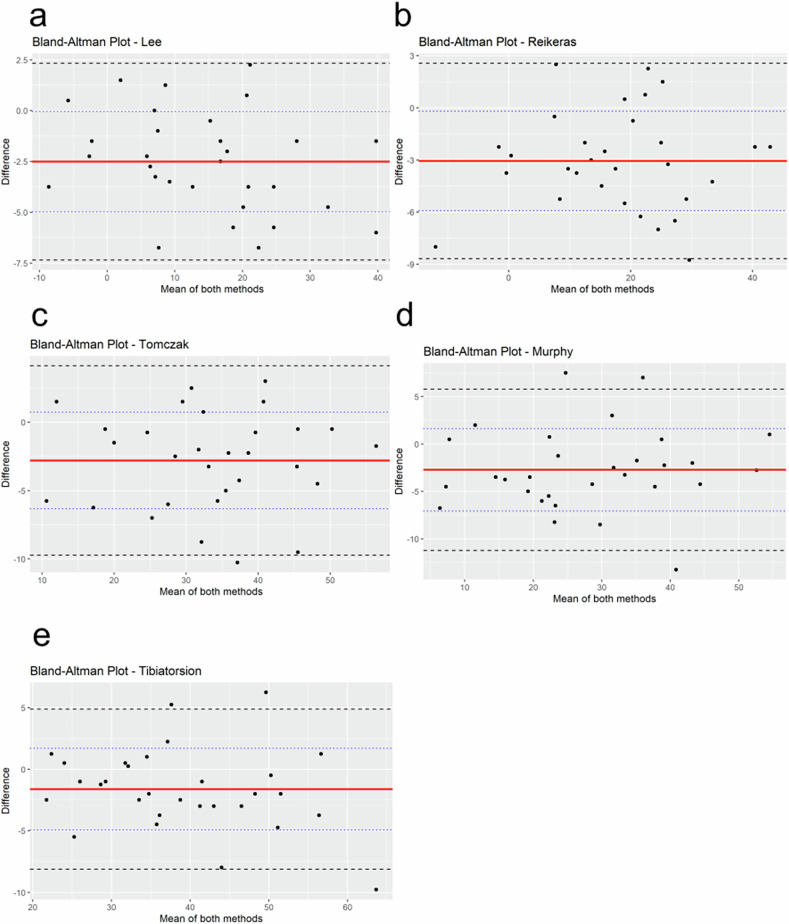
Bland–Altman plots comparing torsion measurements obtained from the dedicated torsion protocol and the FastView localizer for each of the five measurement methods: (**a**) Lee et al [9], (**b**) Reikeras et al [10], (**c**) Tomczak et al [11], (**d**) Murphy et al [3], and (**e**) Tibiatorsion. The *x*-axis displays the mean of both methods (in degrees), while the *y*-axis represents the difference between the two protocols (dedicated protocol *minus* FastView protocol). The solid red line indicates the mean difference (bias). Dashed black lines denote the 95% limits of agreement (mean difference ± 1.96 standard deviations), and dotted blue lines mark the ± 1 standard deviation range. Right and left measurements are combined for each plot. Narrow limits of agreement and consistent bias indicate good agreement between the two protocols

**Fig. 2:**
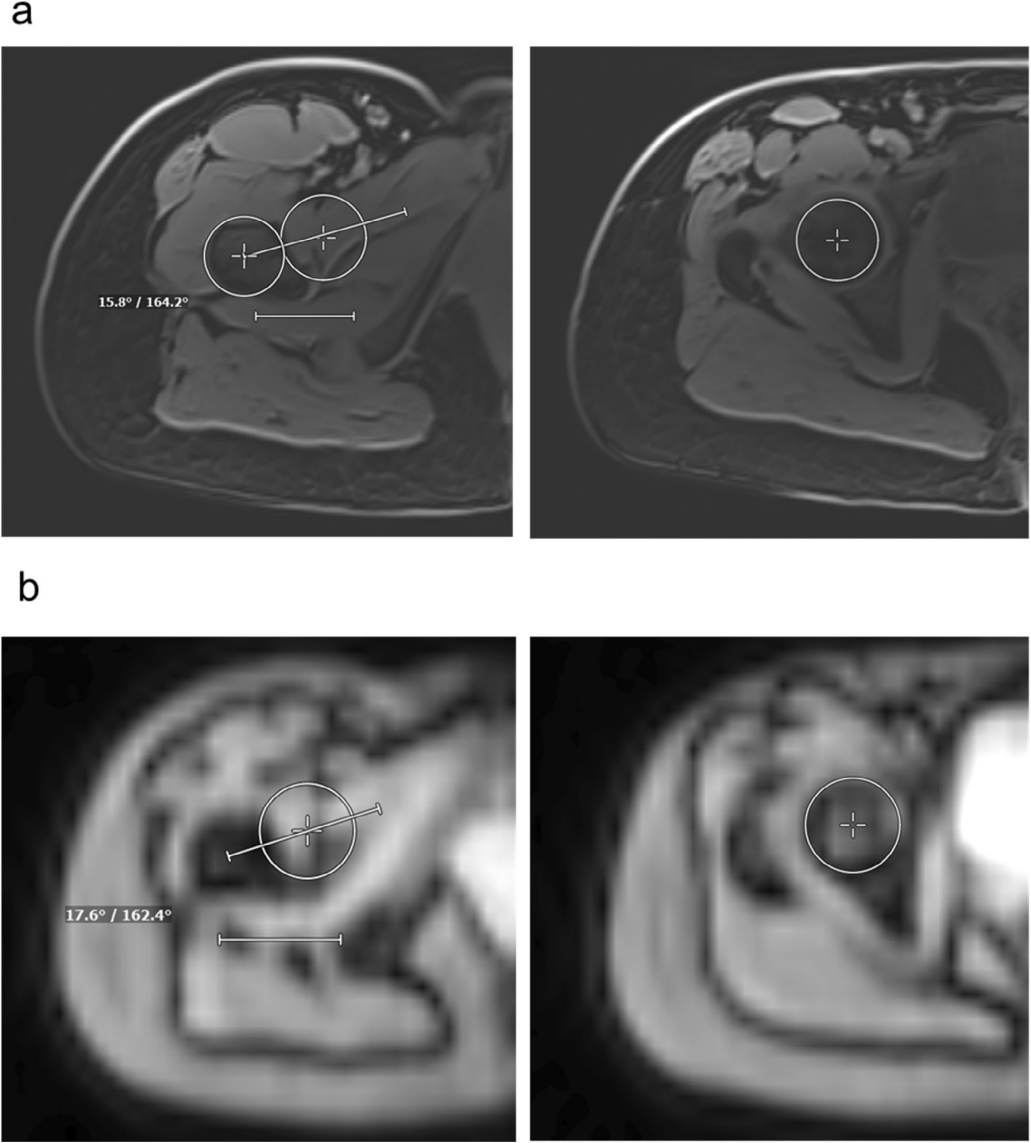
**a** A femoral torsion measurement according to Murphy et al [3], acquired using the dedicated torsion protocol; **b** the corresponding measurement using the FastView localizer. All images originate from the same patient and depict the right hip. This side-by-side comparison demonstrates the visual and quantitative equivalence of both methods in assessing femoral torsion

In total, 60 lower limbs were evaluated in this study. The mean torsion measurements obtained from the dedicated MRI protocol and the FastView localizer did not differ significantly (all *p* > 0.305; Table 2). For femoral torsion, excellent inter-protocol agreement was demonstrated across all four measurement techniques, with ICC ranging from 0.91 to 0.96. The mean differences between the two imaging protocols varied between -2.5° ± 2.5° and -3.1° ± 2.9° (mean ± standard deviation), all of which reached statistical significance (*p* < 0.001). Assessment of tibial torsion showed similarly high concordance, with ICCs ranging from 0.91 to 0.94. The mean difference between the dedicated MRI protocol and the FastView localizer was -1.6° ± 3.3° (*p* = 0.012). Inter-reader agreement was consistently high for all measurements, independent of protocol, with ICCs between 0.95 and 0.99. Bland–Altman analysis further confirmed excellent agreement between both techniques, with *R*² ranging from 0.891 to 0.963 (Figs. 1, 2).

**Table 2:** Comparison of femoral and tibial torsion measurements between the dedicated torsion protocol and FastView

	Femoral torsion				Tibial torsion
	Lee	Reikeras	Tomczak	Murphy	
Dedicated torsion protocol (mean ± SD)	13.2 ± 11.9°	16.3 ± 12.4°	32.1 ± 11.2°	26.6 ± 13.1°	38.3 ± 10.7°
FastView (mean ± SD)	15.7 ± 12.6°	19.3 ± 12.3°	34.9 ± 11.2°	29.4 ± 12.8°	39.9 ± 11.5°
*p*-value	0.424	0.305	0.377	0.384	0.483
Mean difference ± SD	-2.5 ± 2.5°	-3.1 ± 2.9°	-2.8 ± 3.5°	-2.7 ± 4.3°	-1.6 ± 3.3°
95% confidence interval	-3.4 to -1.6°	-4.1 to -2.0°	-4.1 to -1.5°	-4.3 to -1.1°	-2.9 to -0.4°
Pearson *R*^2^	0.963	0.947	0.903	0.891	0.917

The table presents the mean ± standard deviation (SD) of femoral and tibial torsion measurements using the dedicated torsion MRI protocol and the FastView MRI localizer. *p*-values for the comparison between both methods are reported for each measurement technique. The mean difference ± SD, 95% confidence intervals (CI), and Pearson correlation coefficient (*R*²) are also provided for each femoral torsion measurement method—by Lee et al [9], Reikeras et al [10], Tomczak et al [11], Murphy et al [3]—and tibial torsion. High agreement between methods is reflected by strong Pearson correlation values

## Discussion

Our findings indicate that the 3D isotropic FastView MRI localizer provides torsion measurements of the femur and tibia that are highly comparable to those obtained from a dedicated MRI torsion protocol. The high ICCs and strong Pearson correlations underscore the reliability of FastView for torsion assessment. The implementation of FastView can significantly reduce scan times, from nearly 7 ms with the dedicated protocol to approximately 17 s, without compromising measurement accuracy. This efficiency can enhance patient comfort, reduce motion artifacts, and improve workflow in clinical settings.

While statistically significant differences were observed between the dedicated and FastView protocols, these differences remained within clinically acceptable limits. The narrow confidence intervals and strong correlations suggest that these small deviations do not compromise the practical interchangeability of both methods in routine clinical practice.

Of note, the overall preparation time, including patient instruction and positioning, remains largely unchanged. If torsion measurement is the sole clinical indication, these fixed preparation steps may still consume a large part of scanner usage time. However, the use of FastView as a localizer offers a key advantage: it supports both conventional sequence planning for standard lower extremity exams and accurate torsion assessment without additional scan time. This dual functionality can translate into substantial time-savings, especially in dedicated protocols such as those for femoroacetabular impingement, hip dysplasia, or patellar instability. Further, our results align with previous studies demonstrating the feasibility of MRI-based torsion measurements as alternatives to CT, offering the added benefit of eliminating radiation exposure [7]. Moreover, the use of isotropic imaging allows for multiplanar reconstructions, facilitating more flexible and comprehensive assessments.

The retrospective design and single-center setting of this study may limit the generalizability of the findings. In addition, the sample size was relatively small, and further studies with larger cohorts are warranted. Also, no formal power analysis was conducted, as this retrospective study included all eligible patients who underwent both FastView and dedicated MRI within a predefined time window. While this design reflects real-world clinical practice, it inherently limits statistical power estimation and may influence the generalizability of our findings. Future studies with predefined power calculations are warranted to evaluate how FastView integration into broader MRI workflows might reduce total exam duration and streamline resource allocation in busy clinical settings.

In conclusion, the isotropic FastView MRI localizer showed excellent agreement with the dedicated MRI torsion protocol across all femoral and tibial measurements. Strong inter-protocol reliability, narrow limits of agreement, and high correlation values support its validity as an efficient alternative for lower limb torsion assessment. FastView provides a rapid and resource-efficient approach to measuring lower limb torsion and may effectively replace conventional multi-sequence protocols in everyday clinical practice.

## Data Availability

The datasets used and/or analyzed during the current study are available from the corresponding author on reasonable request.

## Abbreviations

3D: Three-dimensional
CT: Computed tomography
ICC: Intraclass correlation coefficient
MRI: Magnetic resonance imaging
